# The Spread of and Death from Infectious Diseases in Sub-Saharan Africa: Implications for FDI Attraction

**DOI:** 10.3390/ijerph192214659

**Published:** 2022-11-08

**Authors:** Kazeem Bello Ajide, Qianxiao Zhang, Ridwan Lanre Ibrahim, Syed Ale Raza Shah

**Affiliations:** 1 Department of Economics, University of Lagos, Yaba 101017, Lagos State, Nigeria; 2 School of Economics & Finance, Xi’an Jiaotong University, Xi’an 710061, China

**Keywords:** foreign direct investment, infectious diseases, policy interventions, system-generalized method of moments, Africa

## Abstract

Are “economic bads” of infectious diseases and “economic goods” of foreign direct investment antagonistic to each other? This is the salient question that this research inquiry unravels for 34 African economies from 2000 to 2017. The empirical evidence revealed the following through a generalized method of moments (SGMM) inter alia: First, the mitigating roles of infectious diseases, such as malaria, HIV prevalence rate and AIDS, on global FDI inflows are unconditionally certified from a statistical and economic sense. Second, the diminishing influences of other confounders, such as low per capita GDP, shallow financial development, excruciating inflationary trend, and natural resource rents curse, are empirically endorsed, on the one hand, while the persistent nature of FDI and trade openness as boosting mechanisms for FDI are unambiguously applauded, on the other hand. Finally, a reduction in the numerical strength of the estimates after accounting for the outliers’ effect from the models and the inclusion of additional controls do not diminish the robustness of already established findings, except for the HIV prevalence rate. On the policy front, if the foreign direct investment is truly pro-development outcomes, any policy interventions that eliminate infectious diseases will be Pareto-improving.

## 1. Introduction

**Figure 1:**
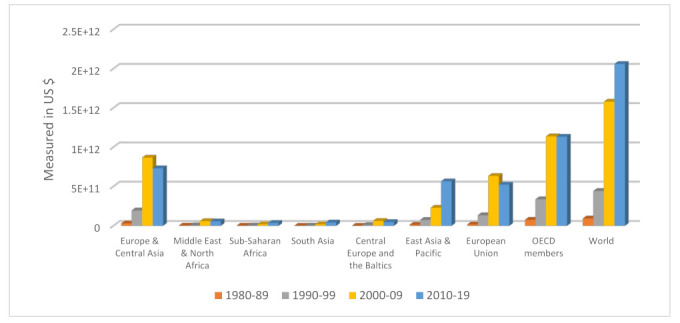
Trend of net FDI inflow across the world regions (in constant US dollars). Source: World Bank Development Indicator Database (2021).

**Figure 2:**
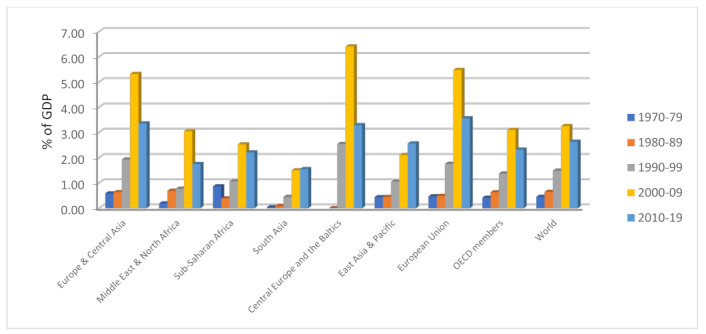
Trend of FDI to GDP across the world regions. Source: World Bank Development Indicator Database (2021).

The socio-economic and politico-institutional impacts of foreign direct investment (FDI) have been well documented in the development literature. In more specific terms, the technological transfers, innovative ideas, and managerial skill spillovers of FDI are arguably the shining features that have accorded FDI a special status among the various types of foreign capital flows for decades [1]. These features and other potential benefits that FDI may confer on the host economy have made its attraction a heightened necessity for virtually all countries worldwide. Of the world continents, Africa appears to be lagging as a destination area for FDI, despite its abundant natural and human resources. The diagrams in Figure 1 and Figure 2 offer some telling evidence of the preceding claims.

As can be observed in Figure 1, Organization of Economic Co-operation and Development (OECD) member countries remain leading destination areas for net FDI inflow in the periods under consideration, while the sub-Saharan (SSA) and South Asia (SA) regions are destination areas with low consideration. Similarly, regarding FDI’s contribution to GDP, SSA appears to have the edge over other regions in 1970–1979. However, this narrative later changed to favor the Middle East and North Africa (MENA) regions in 1980–1989. At the turn of the millennium (2000–2009), Central Europe and the Baltics took over the baton of leadership, directly followed by the European Union. The post-Global Financial Crisis period shows the European Union as a trailblazer concerning FDI’s contribution to the Union’s economic prosperity. What can be inferred from the short exploratory analytic is that SSA is one of the worst performers both in the monetary worth of FDI inflows (measured in constant USD) as well as in FDI’s contribution to the region’s economic progress.

Several reasons have often been cited as responsible for the abysmal performance of SSA as a destination area for FDI in the literature. These factors span economic, socio-cultural, political, institutional, and environmental dimensions. However, little is known about how the health category of the macroeconomy, particularly infectious diseases, has deterred FDI inflow into the region. This is a significant research gap filled by the present inquiry.

Infectious diseases have remained a severe challenge with which SSA has had to contend; diseases such as malaria, tuberculosis (TB), low respiratory infections, diarrheal diseases, and HIV have been rated among the top ten global causes of death. The World Health Organization (WHO) has attributed infectious diseases as representing 32% of global mortality, with 68% and 37% of fatalities claimed by Africa and Southeast Asia, respectively (WHO, 2018). In addition, these infectious diseases equally account for 90% of the global health problems and consequently lead to about 14 million deaths yearly, with 90% of deaths emanating from the developing world. Thus, the pertinent question is: what does this severe incidence of infectious diseases portend for FDI attraction into the region?

The relationship between infectious diseases and foreign direct investment seems salient given their connections' theoretical, anecdotal, and empirical arguments. Taking a theoretical lens, a healthy worker is synonymous with an active and productive workforce [2,3], which in turn correlates with locational decisions on FDI. From an anecdotal perspective, the International Labour Organization (ILO, 2005) also submits that labor supply, quality, and potential productivity are reduced due to infectious diseases, specifically HIV/AIDS; these trends tend to discourage FDI inflow, an essential ingredient of economic development. From an empirical perspective, studies such as [4,5,6,7] have established a positive relationship between good health and FDI inflow. In light of the aforementioned aspects, this study examines the causal linkages between infectious diseases and FDI in the sub-Saharan African region.

More importantly, this study draws its motivations from several considerations. First, in the work of [8], countries were broadly classified into three groups, namely high-income (characterized by high human capital with zero disease incidence), lower income (intermediate human capital with low infectious diseases), and poverty-ridden countries (low human capital with high infectious diseases incidence), based on their stylized facts. It is striking from their study that sub-Saharan African countries largely dominate those in the third group category. In more corroborative terms, the World Malaria report (2021) has shown Africa facing a higher malaria burden than any other continent, recording 96 % of the global deaths from this disease. Similarly, the World Health Organization factsheet equally states TB as the ninth leading cause of global deaths, with over 25% of these deaths claimed by the African region in 2016. In 2019, an estimated 2.5-million-infected TB cases were also found in Africa. For HIV/AIDs, the region has been described as having the most severe cases of HIV and AIDS in the world. In 2015, 36.7 million people were reported to be living with these diseases globally, with SSA claiming about 69.8% (25.6 million). These stylized facts reflect that SSA is an infectious-disease-laden and the most affected region globally. Second, the region is among the least-considered destination areas for FDI, as explained earlier, despite its abundant human and natural resources.

This study makes some novel contributions to the extant literature in the following ways. First, our study analyzes infectious diseases, such as malaria, tuberculosis, and HIV/AIDS, including adults and children newly infected with HIV, on FDI flows for 34 SSA economies from 2000–2017. This makes our work different from that of [9], who only regressed the HIV/AIDS prevalence rate on FDI for SSA between 1990 and 2008. Second, this study controls for econometric problems, which normally distort the interpretation of estimates, thereby leading to wrong policy prescriptions to be drawn. In order to circumvent this, the system's generalized method of moments is employed accordingly. The documented advantages of this estimator lie mainly in its ability to help to resolve problems relating to omitted variable bias, reverse causality, simultaneity, and endogenous issues, and measurement errors.

Overall, this study lends empirical credence to the mitigating impacts of infectious diseases, such as malaria, HIV prevalence rate, and AIDS, on global FDI inflows. This outcome is also robust in accounting for outliers’ effects and inclusion of additional confounding variables together.

In addition to the introductory remarks in Section 1, Section 2 offers some stylized facts about infectious diseases and foreign direct investment in SSA. Section 3 reviews some of the salient literature concerning both theoretical and empirical issues connecting infectious diseases to FDI. Section 4 presents data source, empirical model specification, and estimation techniques. Section 5 analyses the findings with detailed discussions, while Section 6 concludes with policy directions.

## 2. Stylized Facts about Infectious Diseases and FDI Inflow to the SSA Region

This section discusses the behaviors of some key variables of interest to put into perspective the linkages between infectious diseases and FDI across the countries within the sub-Saharan African region. As explained in the introduction, SSA is one of the regions that have had to contend with pervasive infectious diseases and low inflow of FDI among the comity of world regions. SSA surpasses the South Asian region but comes below other regions.

On a country-by-country basis within the region (see Table 1), the FDI contribution to GDP is highest for the Democratic Republic of the Congo with 11.3, while the first and second runners-up are Chad with 7.66 and Sierra Leone with 7.22, respectively. In effect, these contributions of FDI to GDP seems substantial, while Burundi has a minuscule contribution from FDI with a meager ratio of less than one. However, the narrative is not the same when viewing FDI inflows from a monetary value perspective. Nigeria and South Africa averaged USD 4.55 billion and USD 4.15 billion, respectively. Certainly, this is not unexpected as the two are among Africa's top FDI destination areas and SSA in particular. The third position is occupied by Ghana with USD 1.86 billion, with the lowest being occupied by Burundi with USD 100 million. It is worth mentioning that the monetary value of the net FDI inflows to the latter country is negligibly small. Belonging to the category of low FDI recipients are countries such as Guinea Bissau (USD 0.01 billion), Burundi (USD 0.02 billion), Central African Republic (USD 0.03 billion), Gambia (USD 0.05), and Lesotho (USD 0.06). These stylized facts simply point to most economies within the SSA not being destination areas for global FDI inflow.

What can be said about infectious diseases within the region? The country with the highest mortality rate from malaria is Kenya, averaging 25,029.5 within the review period [2,3,6]. The Democratic Republic of the Congo comes after Kenya in the recorded number of malaria deaths. Angola and Tanzania are equally not left behind. Botswana is a nearly malaria-free country in the region, with two-digit figures (13.9). South Africa seems to be topping the mortality scorecard for deaths from TB with a six-digit figure. Countries such as Nigeria (322,755.6), Ethiopia (237,555.6), Democratic Republic of the Congo (203,388.9), and Kenya (200,055.6) are also in that category. In terms of HIV prevalence rate, Botswana and Lesotho are high-risk areas for HIV. Similarly, the place of South Africa and Zimbabwe cannot be overlooked, considering the rate of HIV prevalence in those countries. However, Madagascar and Sudan appear to be the least HIV-afflicted countries in SSA, as their HIV prevalence rate stands at 0.2 each.

Notwithstanding, a sizable number of countries within the region are in the single-digit bound. In addition, deaths from adults newly infected with HIV are more prevalent in South Africa with 376,666.7 (an outlier in the upper bound) and Gambia with 1616.7 (outlier in the lower bound). Nigeria leads in the number of children newly infected with HIV, having an average value of 38,470.6, while South Africa assumes the second position with 38,277.8, and Madagascar claims the lowest position with an annual average of 283.3. For AIDS, South Africa still records the highest number of deaths with an annual mean value of 137,111.1, whereas the least annual average is credited to Gambia with 972.2 deaths in the period under consideration. What is apparent from the foregoing is that countries within the SSA are severely challenged with prevalent infectious diseases coupled with a record of low global FDI inflow. What is unclear is whether the worsening of the region’s infectious diseases can be held responsible for the low FDI inflow.

## 3. Brief Literature Survey

The importance of workers’ health in FDI attraction has been stressed in the World Health Organization’s report of the Commission on Macroeconomics and Health (CMH, 2001). A healthy worker is a source of attraction to foreign investment because of its potential productivity level. Similarly, an unhealthy worker serves as a deterrent to foreign investment due to its associated negative externalities, such as absenteeism and reduced level of productivity, with the consequent effect of raising production costs, thereby deterring FDI from moving into the region. In spite of the supposed linkage between worker’s health status and FDI, it is surprising to note that studies thinking along this dimension are relatively scant.

**Figure 3:**
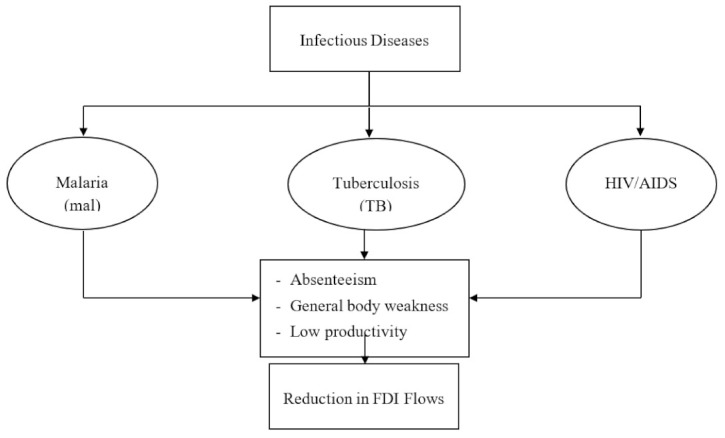
Conceptual linkages between infectious diseases and foreign direct investment inflow.

The health component of human capital is essential to foreign investors because output production requires more than just combining labor and capital. The health status of labor combined with capital resources is highly valued. Thus, the mechanics through which health affects FDI are multi-dimensional. Thus, it is common knowledge that investors would generally prefer more productive workers than unhealthy ones. This is so as healthy workers tend to be more effective. It is also possible that foreign investors may avoid locating their businesses in an environment that is ravaged by infectious diseases. However, if such a decision of locating businesses in a prevalently infectious disease environment occurs, the foreign investors would have to contend with several problems, which include but are not limited to the high cost of conducting business, high staff medical bills, and investing in insurance schemes [1]. More succinctly, Figure 3 presents a conceptual linkage between infectious diseases and foreign direct investment inflow into a country. As can be seen from the diagram, the most global health challenges at present revolve around these three infectious diseases, malaria, tuberculosis, and HIV/AIDS. These diseases often lead to absenteeism and general weakness of the body system, consequently in low productivity from the affected labor force. The presence of workers being ravaged by these infectious diseases is expected to reduce the global FDI into that continent, region, or country, as the case may be. This situation typifies the experience the SSA region might have experienced to date and is still experiencing.

From an empirical perspective, it is pertinent to state that previous empirical studies on the relationship between human capital and FDI largely focused on the education part of human capital resource while ignoring its health status component. Few empirical studies have examined the relationship between health status and FDI. 

Chiappini et al. [10] probed the nexus between FDI and population health in a panel of 143 economies from 1990 to 2019. The study employed the instrumental variable to gauge the empirical relationship. The findings revealed the existence of a positively significant relationship between FDI and health. Similarly, ref. [11] investigated the health effects of FDI in 43 African economies from 1980 to 2016. The findings exposed that FDI improves health outcomes in the sample economies. Furthermore, another essential documented empirical evidence between population health and foreign direct investment was carried out by [4] on 74 industrialized and developing economies from 1980 to 2000. Using a panel data analysis, they found a strong positive significant impact of population health status on the gross inflow of FDI, particularly in low- and middle-income countries. Quantitatively speaking, they state that raising life expectancy by one year would increase FDI by 9% but not without accounting for the role of other confounding factors in their models. The authors of [4,6] further inquired the linkages between communicable diseases and FDI inflow on a broad sample of 114 countries. They employed simple ordinary least-squares (OLS) and two-stage least-squares (TSLS) methods to account for possible endogeneity issues in their models. Their results depicted a small and hardly significant negative influence of infectious diseases on FDI inflow for the former estimator; however, the significant negative impacts become more noticeable when the latter estimator that controls for endogeneity was employed. At about the same period, the study in [9] equally examined the causal relationship between HIV/AIDS and FDI for 41 African economies from 1990 to 2008. The results of the two-step GMM used showed HIV/AIDS as having a deterring effect on FDI for SSA countries and a non-linear impact of HIV/AIDS on FDI with a diminishing impact of the former on the latter as the prevalence rate reduces.

Based on these short empirical reviews, it is apparent that more studies are needed in this direction. Thus, our study adds to this small body of literature by probing into how various infectious diseases (e.g., malaria, tuberculosis, and HIV/AIDS) affect foreign direct investment inflow from the sub-Saharan African region’s perspective.

## 4. Data Source, Model Specification, and Estimation Techniques

### 4.1. Data Source

All variables of interest were obtained from two main sources: the World Health Organization (WHO) and World Development Indicator (WDI). Data involving tuberculosis and malaria were obtained from WHO. The data series about HIV/AIDS, per capita GDP, financial development, inflation, total natural resource rents, and trade openness were from WDI. This study used 34 African economies (Angola, Benin, Botswana, Burkina Faso, Burundi, Cameroon, Central African Republic, Chad, Democratic Republic of the Congo, Congo Republic, Cote d’lvoire, Ethiopia, Gabon, Gambia, Ghana, Guinea, Guinea-Bissau, Kenya, Lesotho, Madagascar, Malawi, Mali, Namibia, Niger, Nigeria, Rwanda, Sierra Leone, South Africa, Sudan, Tanzania, Togo, Uganda, Zambia, and Zimbabwe) for the period 2000–2017. It is worth mentioning that both spatial and temporal dimensions were dictated mainly by data availability consideration.

#### 4.1.1. Main Dependent Variable: Foreign Direct Investment (FDI)

According to OECD, FDI belongs to the category of cross-border investment in which an investor resident in one country, say, United States of America (USA), establishes a lasting interest in and exerts a significant degree of influence over an enterprise resident in another country, say, Nigeria. Alternatively, the ownership of 10% or more of the voting rights in a particular business or enterprise of one country by a foreign investor from another country is evidence of such relationship in the context of conceptualizing FDI. It remains an essential component of international economic integration. It is a route through which technological innovations, know-how, and managerial and entrepreneurial skills are transferred. The World Bank refers to FDI as direct investment equity flows in the reporting country. It is thus a summation of equity capital, reinvestment of earnings, and other capital. Several factors often determine the extent to which these capitals can be attracted into a continent, region, or a country, as the case may be.

#### 4.1.2. Key Regressor Variable: Infectious Diseases

This study considered the major health challenges that the SSA region faces [9,12]. These infectious diseases include malaria, tuberculosis, and HIV (human immunodeficiency viruses)/AIDS (acquired immunodeficiency syndrome). These diseases are often emphasized in all international development goals, such as Millennium Development Goals (MDGs) and Sustainable Development Goals (SDGs). For instance, MDG goal 6 has combatting HIV/AIDS, malaria, and other diseases as one of its prime target goals, while goal 3 of the SDGs has ensuring healthy lives and promoting well-being for all at all ages as one of its driving objectives. These infectious diseases are captured with the number of reported deaths by malaria, the incidence of tuberculosis, prevalence of HIV as a percentage of the total population between ages 15 and 49, number of adults newly infected with HIV (from 15 years and above), number of children newly infected with HIV (between 0–14 years) as well as death rates from AIDS. Each of these infectious diseases was expected to discourage the inflow of FDI into a country. Given the negative externalities of these infectious diseases, a negative relationship was conjectured between them.

#### 4.1.3. Other Regressor Variables

In order to circumvent omitted variable bias, some other important FDI drivers as standard in the empirical literature were paid due consideration. First, per capita GDP is critical factor that foreign investors often consider before making investment decisions. Foreign investors can be easily attracted if the per capita income of the hosting country is high and can become easily discouraged if the per capita GDP is low. There is a one-to-one relationship between per capita GDP and FDI. Studies that accounted for the role of per capita GDP on FDI flows are [7,12,13], among others. Another crucial factor that foreign investors equally place high on their priority list is the level of domestic financial development. A financially sophisticated country tends to attract more foreign investors than a country with shallow financial development such as the SSA region. Thus, a direct positive relationship was unconditionally hypothesized. In addition these driving factors, macroeconomic instability can deter foreign investment from flowing into a country. An often-cited measure of macroeconomic instability in the economic literature is inflation. This refers to a persistent increase in the general prices of goods and services. Foreign direct investment can be deterred from entering a country whose inflation rate is constantly rising. This is so as the persistent rise in prices makes production costs high and unbearable. A situation such as this is commonplace in the African environment whose economies are mainly mono-cultural. Thus, their inability to diversify often attracts a cost, of which inflation forms a substantial part. A negative relationship is presumed between inflation rate and FDI. Deposits of natural resources in a country can motivate foreign investors to relocate to another continent, region, or location, as the case may be. This is often referred to as natural resource-seeking FDI. That is, the location of natural resources could be why FDI is attracted to a particular location. In a case such as this, a direct positive relationship is assumed. Studies such as [7,8,9,14,15] have established a positive relationship between the two. However, this may not be the case if rents from natural resources are seen as a source of wealth. This relationship may be negative between natural resource rents and FDI owing to the natural resource curse that often characterizes the former, particularly in an African environment with weak institutional infrastructure and pervasive rent-seeking behaviors. A negative relationship was also found by [16,17], among others. Similarly, the role of and the degree of trade openness largely determines the extent to which FDI can be attracted into a country. For a country that is fully open to the outside world, a higher FDI is expected and vice versa. Aside from the preceding factors, the path-dependent nature of FDI equally matters. That is, a previous experience of FDI could easily deprive the present FDI experience. Studies such as [18,19] account for the persistent nature of FDI. Thus, a positive nexus was intuitively envisaged. Moreover, the variable description is shown in Appendix A.

### 4.2. Model Specification and Discussion on the Estimation Techniques

In line with previous empirics on both determinants [20,21,22,23] and deterrent factors of FDI [21], the empirical model for unravelling the link between infectious diseases and FDI is as follows:

(1) $$fdi_{it}=\sigma_{0}+\beta_{1}\inf ectdis_{it}+\beta_{2}othercontr_{it}+\varepsilon_{it}$$

where $fdi_{}$ is foreign direct investment and infectdis represents different type of infectious diseases such as malaria (mal), tuberculosis (tb), HIV prevalence rate (human immunodeficiency viruses), and AIDs (acquired immunodeficiency syndrome), respectively. othercontr stands for other control variables, including per capita GDP, financial development, inflation, total natural resource rents to GDP, and trade openness. σ is the intercept (constant term) of the estimated models, while $\beta_{1}-\beta_{2}$ are the parameter estimates of the models. i, t, and ε are the country, time periods, and the error-term white noise in that order, respectively.

Thus, Equation (1) can be rewritten as

(2) $$fdi_{it}=\sigma_{0}+\beta_{1}\inf ectdis_{it}+\beta_{2}\sum_{i=1}^{5}othercontr_{it}+\varepsilon_{it}$$

Equation (2) can be empirically expanded thusly:

(3) $$fdi_{it}=\sigma_{0}+\beta_{1}{\left(\begin{array}{l} \inf ectdis_{it}=\\ malaria(mal)\\ tuberculosis(tb)\\ HIV/AIDS \end{array}\right)}_{it}+\beta_{2}pgdp_{it}+\beta_{3}fin_{it}+\beta_{4}\inf_{it}+\beta_{5}totnat_{it}+\beta_{6}trade_{it}+\varepsilon_{it}$$

where fdi,pgdp,fin,inf,totnat, and trade are a foreign direct investment, financial development, inflation, total natural resource rents, and trade openness, respectively.

Investigating the relationship between FDI and many of these confounding variables as specified in the empirical model above (particularly Equation 3) raised some concerns about endogeneity issues. To resolve these issues, the equation must be estimated using a dynamic generalized method of moments (GMM) based on the Arellano–Bond (AB, hereafter) methodology.

The AB methodology specifies a dynamic model that allows control for time-invariant country-specific effects. This is considered appropriate, particularly concerning FDI, where variables such as institutions, political regime, ethnicity, and geography hardly display variations over time. Thus, we rewrote the above equations in a more general form as

(4) $$y_{it}=\sigma +\alpha y_{i,t-1}+X_{i,t}^{\prime }\beta +\mu_{i}+\nu_{i,t}$$

where y is the ratio of FDI to GDP and X is the vector of infectious diseases and other confounding variables determining FDI in a country, region, or continent as the case may be. μ denotes the time-invariant country-specific effects. To remove these effects, the first difference of Equation (4) was taken to achieve the following equation

(5) $$y_{it}-y_{i,t-1}=\sigma +\alpha (y_{i,t-1}-y_{i,t-2})+(X_{i,t}^{\prime }-X_{i,t-1}^{\prime })\beta +(\nu_{i,t}-\nu_{i,t-1})$$

There was a need to control for possible endogeneity concerns between the explanatory variables and the main outcome variable, which in this case is denoted by $y_{i,t}$. Then, the equation can be estimated using as instruments both the lagged values of the right- and left-hand side variables in levels. The generated instruments were considered as valid provided the error term (ν) was not serially correlated. Due to some statistical limitations of estimating the above instrumental variable at least in a small sample of path dependency nature (that is, persistent explanatory variables), using lagged levels was inefficient and weak, mainly when run in differences. In order to rectify this econometric weakness, Blundell and Bond (BB) developed the system-generalized method of moments (SGMM) by combining the regression in the first difference using both lagged levels and differences as instruments.

### 4.3. Analysis of Descriptive Statistics

Table 2 and Table 3 present the basic summary statistics of variables used in the estimations. The average value of net foreign direct investment inflow for the region was less than USD 1 billion between 2000 and 2017, while the maximum and minimum values stand at USD 10 billion (Angola in 2015) and USD 7.4 billion deficit (Angola in 2017). In addition, the average FDI contribution to economic activities is 3.45, with the maximum and minimum being 46.28 (Chad in 2002) and −6.06 (Angola in 2017), respectively. The deficit in the flow of FDI into Angola may be responsible for the negative FDI contribution to growth in that country. The five years of economic recession witnessed by Angola beginning in 2016 may be held accountable for the country’s abysmal performance as a destination area for FDI in that year. The economic recession may be a reason why the Angolan economy dropped from its position as the third largest economy to eighth in 2020. For the infectious diseases, malaria, tuberculosis, HIV prevalence, and AIDS averaged 3619.95, 70,064.80, 5.78, and 23,703.27, in that order. The mean value of tuberculosis is far higher than those of other infectious diseases in the period under consideration (2000–2017). It is equally important to mention that the mean value of the number of adults infected with HIV is higher than that of the children, as seen in the table. Other statistics relating to other confounding variables determining FDI can be observed in the table. Table 3 shows the correlation matrix of all variables of interest. Quite interestingly, all the variables of interest, except for natural resource rents and trade openness, are negatively correlated with foreign direct investment.

**Figure 4:**
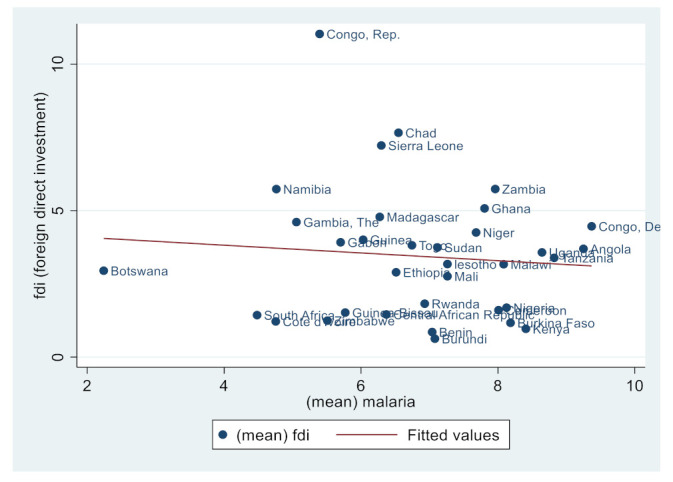
Malaria and FDI.

**Figure 5:**
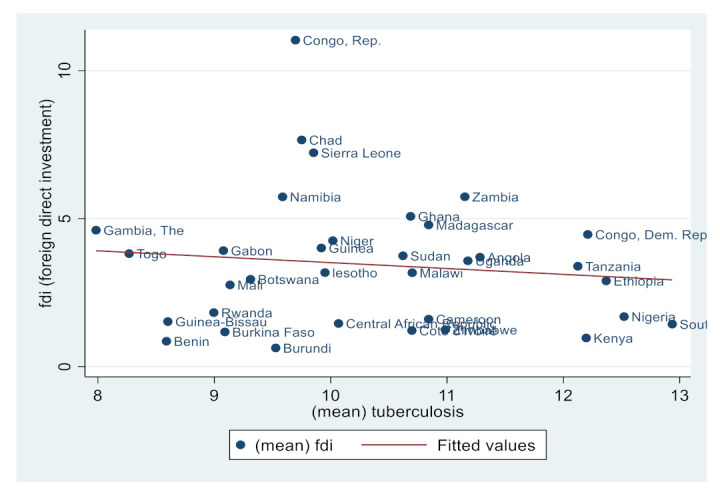
Tuberculosis and FDI.

**Figure 6:**
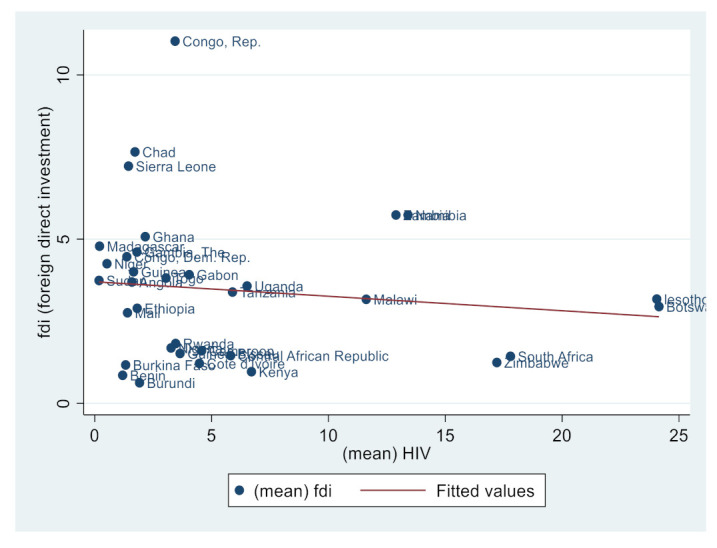
HIV prevalence and FDI.

**Figure 7:**
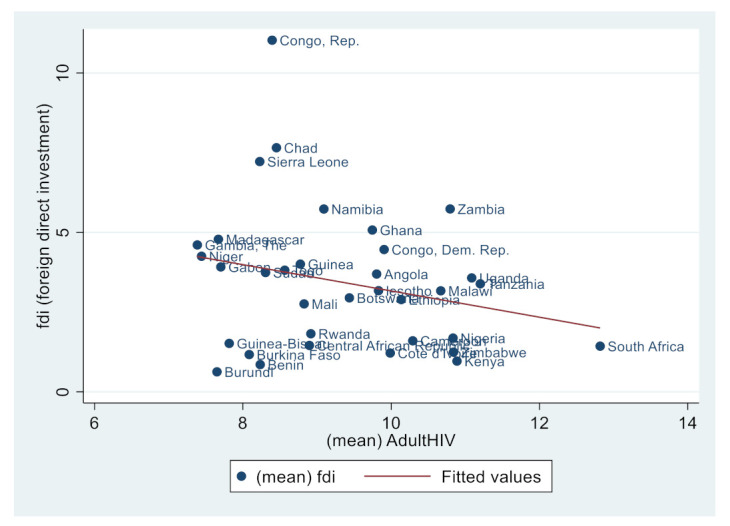
Adults newly infected with HIV and FDI.

**Figure 8:**
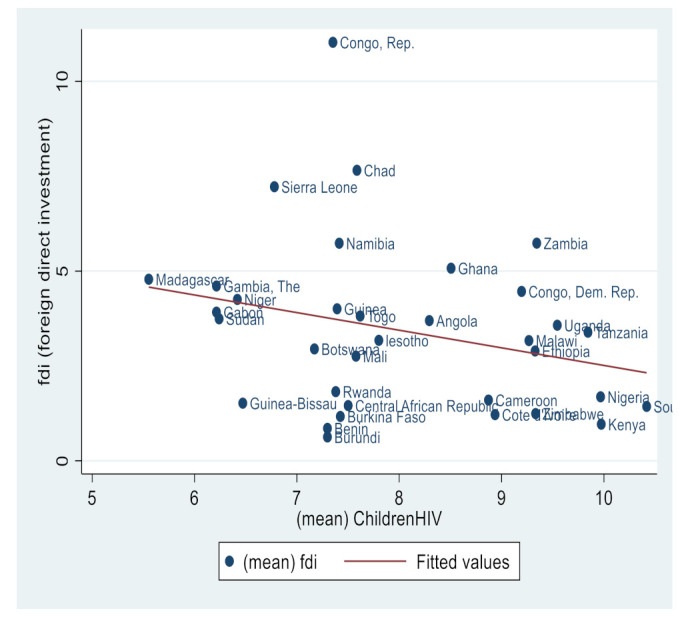
Children newly infected with HIV and FDI.

**Figure 9:**
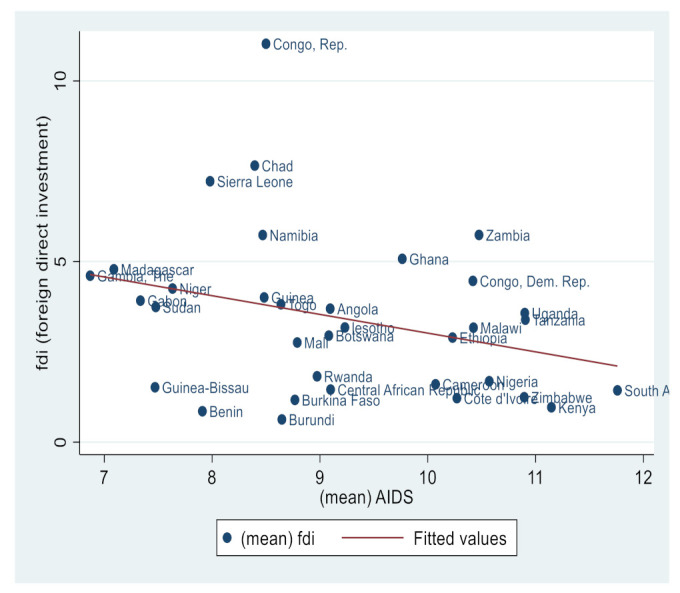
AIDS and FDI.

Similarly, Figure 2, Figure 3, Figure 4, Figure 5, Figure 6 and Figure 7 depict the nature of relationship between each of the main variables (including malaria, tuberculosis, HIV prevalence rate, number of adults newly infected with HIV, and number of children newly infected with HIV and AIDS) and FDI. Figure 4, Figure 5, Figure 6, Figure 7, Figure 8 and Figure 9 depict bivariate relationships between malaria and FDI, tuberculosis and FDI, HIV and FDI, adults newly infected with HIV and FDI, children newly infected with HIV and FDI, and AIDS and FDI, respectively.

It is interesting to note that all infectious diseases maintain a negative relationship with FDI as depicted by the negative slope of each diagram. By implication, the higher the infectious diseases, the lower the inflow of FDI into the region. Notwithstanding the observed negative slopes in these relationships, the pattern of slopes is not uniform across the connections. For instance, Figure 5, Figure 6 and Figure 7 appear steeper than Figure 2, Figure 3 and Figure 4, which are relatively flattering in slopes. From Figure 2, it is apparent that virtually all the countries are malaria-laden, thus deterring foreign investors. The only moderate case is the Congo Republic, as observed in the diagram. However, it is surprising that an outlier country such as Botswana has a reduced number of malaria deaths and still attracts lower FDI inflows. In Figure 3, African countries are not as concentrated as observed in the case of malaria. Despite this, the attraction of FDI is still much lower. The outstanding exception is still the Congo Republic with a moderate case. However, countries such as Nigeria, Kenya, Ethiopia, Tanzania, Democratic Republic of the Congo, and South Africa are comparatively worse as destination areas for FDI, as occasioned by the rate of mortality resulting from TB. In Figure 4, HIV seems more prevalent in countries such as Lesotho, Botswana, Zimbabwe, and South Africa. As such, the FDI contribution to their economic growth is correspondingly low. The exceptional countries in this respect are the Congo Republic, Chad, and Sierra Leone, with limited HIV prevalent cases with higher FDI contributions to their respective economic progress. South Africa is an outlier country with higher cases of adults newly infected with HIV, and this has correspondingly reduced the country’s FDI to economic growth ratio. The Congo Republic retains its special performance status (see Figure 4 for visual confirmation). In Figure 6, there are more children newly infected with HIV in countries such as South Africa, Nigeria, Kenya, Tanzania, and Zimbabwe. Contrarily, countries such as Madagascar, Gambia, Nigeria, and Gabon have comparatively lower cases. For AIDS, South Africa takes the lead, while at the same time experiencing a declining FDI to growth ratio, as can be observed in Figure 7.

Against the preceding background, it can thus be inferred that there seems to be a significant negative correlation between infectious diseases in varying degrees and the inflow of FDI into the region. However, the observed negative correlations do not suggest, in principle, causality. Consequently, the need to conduct further investigation merits empirical attention but not without accounting for other FDI confounders.

## 5. Analysis of Empirical Findings and the Associated Discussions

Table 4 presents substantive results of the impacts of infectious diseases on FDI in the SSA region. It is interesting to state that all infectious disease measures conform with theoretical priors across the estimated FDI models. This finding is broadly aligned with the outcomes of the exploratory analytics presented in Figure 2, Figure 3, Figure 4, Figure 5, Figure 6 and Figure 7 and that of the correlation matrix in Table 3. This suggests that, at higher levels of these various infectious diseases, less FDI is attracted into the region. Notwithstanding the observed conformity in theoretical priors, on the one hand, the magnitude of statistical impacts only favors three out of the six measures of infectious diseases. The favored measures include malaria, HIV prevalence rate, and AIDS. By implication, except tuberculosis, adults newly infected with HIV and children newly infected with HIV are not statistically significant in the region but are dully aligned with the theoretical expectations. Taking the variables of the infectious diseases that are statistically significant in turn, a 10-unit increase in malaria deaths would lead to a 48.7 reduction in FDI, whereas for the prevalent HIV cases, a 10-unit increase would only result in a 39.3 decrease in FDI. Similarly, such a 10-unit increase in AIDS would lessen FDI by 37.7. These findings can reasonably be linked to the associated negative externalities that often come with these infectious diseases. For instance, malaria-infected workers, HIV-infected workers, AIDS-afflicted labor, absenteeism, high worker turnover, and low productivity would tend to limit the inflow of foreign workers into the region. The reasons for this may not be implausible. First, production costs will tend to increase as resources will be channeled to patients afflicted with these diseases. Second, potential foreign investors risk contracting these diseases in high-risk infectious areas. Third, the chances of making abnormal or normal profits are slim and thin more often than not, which usually form the main overriding goal of foreign investors investing abroad. This seems so, as such resources would have been channeled toward recuperating workers from these infectious diseases.

The impacts of other explanatory variables are equally notable. To begin with, the log of per capita GDP does not confirm a priori expectations and is statistically relevant for the most part at the 1% level across the specifications. However, this sounds counterintuitive but seems plausible for the SSA region’s case. Why could this be? This finding is possible in an African environment in general and the SSA region in particular, where per capita GDP is low compared to advanced nations with higher GDP per capita. Looking at Table 4, the per capita GDP in the region stands at USD 1529.36, with upper and lower values of USD 7888.06 and 258.63 for the lower-middle-income and low-income countries, respectively. In effect, a low per capita GDP has a discouraging impact on attracting FDI into the region. This finding is consistent with [9,13,19]. Financial development also has a significant negative impact on FDI. This may be explained by the fact that African financial development is still in its infancy. Accessing financial services or credits constitutes a more significant concern for most people in the region. This can be gleaned from an average of 16.38 for the recognition extended to the private sector. Macroeconomic instability is another major source of concern for FDI attraction, as can be observed from the negative coefficient on the inflation variable. It is found to be statistically significant at the 1% conventional levels. Intriguingly, the coefficient of the total natural resource rents has an equally negative value and was found to be relevant at the peak of statistical significance. This is counterintuitive, in that natural resources are considered bait to attract FDI into the country; however, it is deterring it in this case. This is plausible in the SSA’s case, where rents from natural resources are already seen as a source of wealth. The negative coefficient on natural resource rents lends further supportive empirical evidence to the natural resource curse thesis characterizing the region. However, this observed outcome is not in tandem with [9]. However, ref. [23] has lent empirical credence to the region’s resource curse argument, particularly regarding economic complexity. Additionally, we can ignore the role of trade openness as one key determining factor of FDI. In this case, trade openness constitutes a pulling pad of FDI since it complies with the hypothesized sign and is statistically significant at the 1% level. The path-dependent nature of FDI on the present FDI has an equal worth according to empirical commendation.

Despite the economic and statistical usefulness of the variables of interest for the most part on FDI in the region, the performance of some basic diagnostics deserves some attention as well. The statistical insignificance of AR(2) is a reflection of the absence of autocorrelations in the estimated models, while the non-statistical significance of the Hansen tests is a clear testimony of the validity of the internal instruments used in the models.

### 5.1. Robustness Checks

Table 5 presents the empirical outcomes of the causal linkage between infectious diseases and FDI by removing some outliers from the sample of countries used. Interestingly, malaria still enters negatively into FDI models and is statistically significant at the 1% level. However, unlike Table 4, the magnitude of the impact of malaria deaths on FDI reduces from −0.487 to −0.134, confirming the greater impacts of the outlier countries on the estimates. Tuberculosis fails, on two fronts, to pose a threat to FDI. First, it does not align with the theoretical expectations. Second, it is statistically redundant, as reported in Table 4. HIV prevalence is positive and statistically significant at the 10% level. The positive sign on the HIV coefficient runs contrary to expectations. This outcome implies that the prevalence of HIV in low-risk countries does not deter the inflow of FDI since the outlier countries (high-risk) were removed from the sample. Adults newly infected with HIV exert a reducing effect on FDI, occurring at the 10% significance level. However, children newly infected with HIV do not pose any risk to foreign investors. The possible explanation for this may be linked to the fact that children are not eligible to work, unlike their adult counterparts. Thus, the chances of them linking up with foreigners investing in abroad are slim. Lastly, AIDS carries the expected prior and has statistically weight on FDI inflow into the region, as indicated by its 1% significance level. In terms of other covariates, the hypothesized signs on variables, such as financial development, inflation, total natural resource rents, trade openness, and lagged value of FDI, are in tandem with previously the established findings in Table 4, except for the log of per capita GDP whose hypothesized priors are, for the most part, positive, while the magnitude of statistical relevance oscillates between 1% and 10% levels, respectively.

### 5.2. Accounting for other Important FDI Determinants (Results Are Available upon Special Request)

We equally controlled for other important drivers of FDI, including political stability, government expenditure, infrastructural facilities, and real exchange rates. The findings do not look significantly different from the conclusions established in Table 4.

## 6. Conclusions and Policy Recommendations

Attracting foreign direct investment has always remained a top priority in the development policy agenda of most economies, particularly those in the developing world. This has always been due to their severe resource constraints and low-income levels. Consequently, extensive research has been conducted to unravel the possible determinants and deterrents of FDI inflows into a country, region, or continent, as the case may be. Of the plethora of determinant and deterrent factors identified to date, little or almost nothing is known about how infectious diseases have impacted the global flows of FDI into the African continent in particular. In light of this open gap in the literature, this paper added to a small but emerging strand of knowledge in the infectious diseases–FDI nexus for 34 economies within the African continent, where both ‘’economic bads’’ and ‘’economic goods’’ exist over the period 2000–2017. Using the system-generalized method of moments, the empirical estimations show inter alia: First, the mitigating roles of infectious diseases, such as malaria, HIV prevalence rate, and AIDS, on global FDI inflows are unconditionally certified both in statistical and economic senses. Second, the diminishing influences of other confounders, such as low per capita GDP, shallow financial development, excruciating inflationary trend, and natural resource rents curse, are empirically endorsed, on the one hand, while the persistent nature of FDI and trade openness as boosting mechanisms for FDI are unambiguously applauded, on the other hand. Finally, the reduction in the numerical strength of the estimates after accounting for the outliers’ effect from the models and the inclusion of additional controls do not diminish the robustness of the already established findings, except for the exception of HIV prevalence rate.

In light of the development outcome impacts of foreign direct investment, tackling infectious diseases through their complete extermination seems sacrosanct and non-negotiable. This can be achieved by allocating an adequate budget to the health sector of the economy. More importantly, all preventive measures laid out for treating malaria, HIV, and AIDS should be complied with in accordance with the stated medication solutions. Additionally, the environmental hazards contributed by multinationals are a major source of health deterioration in Africa. More should be achieved by governments to improve environmental regulations to halt the hazardous impacts of the production activities by multinationals. Health practitioners also have significant roles to play by providing the policy advice needed to governments to ensure preventive measures are taken to safeguard the life of every citizen across the African countries.

Despite the novelty of the present study, some important areas of research are not covered. For instance, the role of regulatory quality in modulating between FDI and infectious diseases is not addressed. Hence, future studies can explore this avenue.

## Figures and Tables

**Table 1:** Annual averages of infectious diseases and FDI of countries within the SSA region.

Country	Foreign Direct Investment (% of GDP)	Foreign Direct Investment (USD Billion)	Malaria	Tuberculosis	HIV Prevalence Rate	Adults Newly Infected with HIV	Children Newly Infected with HIV	AIDS
Angola	3.70	0.19	11,657.7	81,722.2	1.6	18,333.3	4116.7	9166.7
Benin	0.86	0.10	1305.2	5816.1	1.2	3783.3	1669.4	2844.4
Botswana	2.95	0.30	13.9	11,461.1	24.1	13,066.7	1628.3	9750.0
Burkina Faso	1.17	0.13	6019.8	8883.3	1.3	3438.9	1957.2	7116.7
Burundi	0.63	0.02	1860.2	13,888.9	1.9	2283.3	1669.4	6466.7
Cameroon	1.60	0.42	3520.5	51,166.7	4.6	30,166.7	7355.6	23,777.8
Central African Republic	1.46	0.03	1064.2	24,666.7	5.8	7516.7	1943.9	9288.9
Chad	7.66	0.28	1025.8	17,444.4	1.7	4705.6	2022.2	4650.0
Congo, Dem. Rep.	4.46	1.03	18,252.9	203,388.9	1.4	20,722.2	10,588.9	34,722.2
Congo, Rep.	11.03	1.24	642.5	16,444.4	3.4	4427.8	1561.1	5033.3
Cote d'Ivoire	1.22	0.39	3694.9	45,000.0	4.5	22,444.4	8094.4	30,388.9
Ethiopia	2.89	1.02	1218.5	237,555.6	1.8	25,722.2	13,155.6	33,833.3
Gabon	3.92	0.54	478.8	8855.6	4.0	2227.8	500.0	1555.6
Gambia	4.61	0.05	243.2	2955.6	1.8	1616.7	500.0	972.2
Ghana	5.08	1.86	2756.1	43,777.8	2.2	17,111.1	5011.1	17,722.2
Guinea	4.01	0.28	659.1	20,333.3	1.7	6511.1	1638.9	4927.8
Guinea-Bissau	1.52	0.01	487.8	5522.2	3.7	2500.0	656.7	1777.8
Kenya	0.97	0.49	25,029.5	200,055.6	6.7	54,888.9	25,088.9	79,388.9
Lesotho	3.17	0.06	1552.8	21,222.22	24.1	18,944.4	2716.7	10,927.8
Madagascar	4.79	0.48	551.9	51,277.8	0.2	2311.1	283.3	1238.9
Malawi	3.17	0.18	5491.6	45,333.3	11.6	43,333.3	11,422.2	39,444.4
Mali	2.76	0.27	1552.8	9288.9	1.4	6905.6	1955.6	6677.8
Namibia	5.73	0.51	483.5	14,833.3	13.4	9144.4	1901.7	5488.9
Niger	4.25	0.37	2273.6	33,222.2	0.5	1816.7	623.3	2166.7
Nigeria	1.69	4.55	6331.2	322,755.6	3.3	96,000.0	38,470.6	74,117.6
Rwanda	1.82	0.12	1454.0	8135.3	3.5	8094.4	1961.1	9905.6
Sierra Leone	7.22	0.23	1790.4	19,222.2	1.4	3744.4	912.8	2955.6
South Africa	1.43	4.15	113.3	421,277.8	17.8	376,666.7	38,277.8	137,111.1
Sudan	3.74	1.43	1355.2	41,388.9	0.2	4094.4	513.3	1900.0
Tanzania	3.39	1.00	10,173.3	184,944.4	5.9	73,388.9	19,777.8	60,000.0
Togo	3.82	0.12	1311.6	3977.8	3.1	5338.9	2127.8	5727.8
Uganda	3.57	0.63	5911.8	71,888.9	6.5	65,611.1	14,761.1	58,166.7
Zambia	5.74	0.93	5260.5	70,000.0	12.9	49,000.0	12,100.0	40,777.8
Zimbabwe	1.24	0.17	690.6	61,055.6	17.2	53,055.6	13,250.0	68,722.2

Source: authors’ computation.

**Table 2:** Descriptive statistics of all variables of interest.

Variable	Mean	Std. Dev.	Min	Max	Obs
fdi	3.45	4.67	−6.06	46.28	612
fdiv	6.93 × 10^8^	1.48 × 10^9^	−7.40 × 10^9^	1.00 × 10^10^	612
mal	3619.95	6812.10	3.00	51,842.00	562
logmal	6.780	2.170	0.000	10.856	586
tb	70,064.80	100,902.80	590.00	493,000.00	611
logtb	10.319	1.304	6.380	13.108	611
phiv	5.78	6.56	0.10	27.00	611
adhiv	31,038.46	66,125.02	1000.00	500,000.00	611
logadhiv	9.315	1.407	0.000	13.122	612
chhiv	7308.30	10,844.80	200.00	64,000.00	611
logchhiv	7.986	1.402	0.000	11.067	612
aids	23,703.27	34,195.59	500.00	210,000.00	611
logaids	9.17	1.42	0.00	12.25	612
pgdp	1529.36	1728.09	258.63	7888.06	612
fin	16.38	21.58	0.00	142.42	545
inf	13.88	107.98	−21.17	2630.12	612
totnat	12.31	10.61	0.59	58.69	612
trade	62.64	28.18	1.38	165.06	576

**Table 3:** Correlation matrix.

	fdi	mal	Tb	phiv	adhiv	chhiv	aids	lpgdp	fin	inf	totnat	trade
fdi	1.00											
mal	−0.03	1.00										
tb	−0.05	0.23	1.00									
phiv	−0.10	−0.38	0.20	1.00								
adhiv	−0.14	0.06	0.79	0.53	1.00							
chhiv	−0.14	0.19	0.74	0.32	0.92	1.00						
laids	−0.17	0.14	0.75	0.42	0.93	0.97	1.00					
lpgdp	−0.01	−0.34	0.23	0.54	0.41	0.22	0.24	1.00				
fin	−0.11	−0.21	0.36	0.51	0.54	0.38	0.42	0.50	1.00			
inf	−0.03	0.04	0.07	−0.03	0.04	0.06	0.06	−0.06	−0.04	1.00		
totnat	0.18	0.09	−0.06	−0.30	−0.25	−0.13	−0.17	0.00	−0.24	−0.01	1.00	
trade	0.34	−0.19	−0.16	0.42	−0.01	−0.08	−0.06	0.33	0.04	−0.06	0.26	1.00

**Table 4:** Empirical estimates of the relationship between infectious diseases and FDI (using GMM).

Variables	(Model 1)	(Model 2)	(Model 3)	(Model 4)	(Model 5)	(Model 6)
Dependent Variable: Foreign Direct Investment (FDI)						
L.fdi	0.307 ***	0.413 ***	0.405 ***	0.408 ***	0.427 ***	0.377 ***
	(0.045)	(0.030)	(0.032)	(0.029)	(0.037)	(0.036)
mal	−0.487 ***					
	(0.084)					
phiv		−0.393 ***				
		(0.054)				
tb			−0.002			
			(0.164)			
adhiv				−0.277		
				(0.254)		
chhiv					−0.146	
					(0.218)	
Aids						−0.377 **
						(0.148)
lpgdp	−2.076 ***	−1.041 ***	−1.177 **	−0.947 **	−0.885 *	−1.731 ***
	(0.513)	(0.259)	(0.490)	(0.449)	(0.518)	(0.401)
fin	−0.079 **	0.029 **	−0.001	−0.070 ***	−0.068 ***	−0.022 *
	(0.031)	(0.011)	(0.018)	(0.024)	(0.021)	(0.011)
inf	−0.032 ***	−0.017 ***	−0.016 ***	−0.011 ***	−0.014 ***	−0.015 ***
	(0.004)	(0.003)	(0.002)	(0.003)	(0.003)	(0.003)
totnat	−0.170 ***	−0.097 ***	−0.076 ***	−0.123 ***	−0.121 ***	−0.099 ***
	(0.033)	(0.022)	(0.023)	(0.032)	(0.029)	(0.026)
trade	0.173 ***	0.088 ***	0.095 ***	0.111 ***	0.101 ***	0.105 ***
	(0.016)	(0.012)	(0.013)	(0.013)	(0.012)	(0.010)
Constant	13.330 ***	6.705 ***	5.482	7.268	6.059	12.894 ***
	(3.636)	(1.820)	(4.355)	(4.707)	(4.655)	(3.754)
**Other diagnostics**						
Observations	469	484	484	485	485	485
Number of c_id	32	32	32	32	32	32
AR(1)	0.102	0.078	0.078	0.077	0.071	0.085
AR(2)	0.337	0.223	0.222	0.221	0.213	0.235
Hansen test	0.259	0.287	0.440	0.463	0.465	0.375
Number of Instruments	28	28	28	28	28	28

Standard errors in parentheses. *** *p* < 0.01, ** *p* < 0.05, * *p* < 0.1.

**Table 5:** Empirical estimates of the relationship between infectious diseases and FDI (removing outliers).

Variables	(Model 1)	(Model 2)	(Model 3)	(Model 4)	(Model 5)	(Model 6)
Dependent Variable: Foreign Direct Investment (FDI)						
L.fdi	0.534 ***	0.539 ***	0.483 ***	0.524 ***	0.491 ***	0.512 ***
	(0.027)	(0.016)	(0.024)	(0.016)	(0.020)	(0.020)
mal	−0.134 ***					
	(0.044)					
tb		0.211				
		(0.158)				
phiv			0.214 *			
			(0.120)			
adhiv				−0.218 *		
				(0.112)		
chhiv					−0.262	
					(0.277)	
aids						−0.207 ***
						(0.027)
lpgdp	0.256	−0.825 ***	0.657 *	0.702 *	0.144	0.285
	(0.187)	(0.193)	(0.322)	(0.353)	(0.272)	(0.414)
fin	−0.058 ***	−0.040 *	−0.058 **	−0.113 ***	−0.125 ***	−0.100 ***
	(0.011)	(0.023)	(0.027)	(0.029)	(0.034)	(0.024)
inf	−0.006	−0.041 ***	0.001	−0.022 ***	−0.026 ***	−0.024 ***
	(0.005)	(0.004)	(0.003)	(0.003)	(0.004)	(0.003)
totnat	−0.177 ***	−0.113 ***	−0.141 ***	−0.100 ***	−0.128 ***	−0.086 ***
	(0.030)	(0.030)	(0.029)	(0.027)	(0.033)	(0.020)
trade	0.126 ***	0.119 ***	0.168 ***	0.137 ***	0.155 ***	0.126 ***
	(0.008)	(0.006)	(0.011)	(0.013)	(0.010)	(0.011)
Constant	−3.544 **	0.283	−9.811 ***	−6.282 **	−2.832	−3.314
	(1.293)	(2.383)	(2.245)	(3.002)	(2.293)	(2.961)
**Other diagnostics**						
Observations	424	384	408	451	403	451
Number of c_id	29	26	27	30	27	30
AR(1)	0.005	0.004	0.003	0.002	0.003	0.002
AR(2)	0.596	0.839	0.450	0.405	0.369	0.400
Hansen test	0.233	0.720	0.624	0.484	0.562	0.374
Number of Instruments	28	28	28	28	28	28

Standard errors in parentheses. *** *p* < 0.01, ** *p* < 0.05, * *p* < 0.1.

## Data Availability

Data are available upon special request.

## Appendix A

**Table A1:** Description of Variables.

Variables	Signs	Measurements	Data Sources
Foreign direct investment	fdi	Foreign direct investment, net inflows as a percentage of GDP	WDI
malaria	mal	Number of malaria deaths	World Health Organization (WHO)
tuberculosis	tb	The incidence of TB (tuberculosis)	World Health Organization (WHO)
HIV prevalence rate	phiv	Prevalence of HIV (% of total population aged 15–49)	WDI
Adults newly infected with HIV	adhiv	Number of adults (ages 0–14) newly infected with HIV	WDI
Children newly infected with HIV	chhiv	Number of children (aged 15+) newly infected with HIV	WDI
AIDS	aids		WDI
Per capita GDP	pgdp	GDP per capita (constant 2015 in USD)	WDI
Financial development	fin	Domestic credit to private sector as a percentage of GDP	WDI
Inflation	inf	Inflation, GDP deflator (annual %)	WDI
Total natural resource rents	totnat	Total natural resources rent as a percentage of GDP	WDI
Trade openness	trade	Trade (export plus import) as a percentage of GDP	WDI
